# Correction: Beylerli et al. MiRNAs as Noninvasive Biomarkers and Therapeutic Agents of Pituitary Adenomas. *Int. J. Mol. Sci.* 2020, *21*, 7287

**DOI:** 10.3390/ijms26104608

**Published:** 2025-05-12

**Authors:** Ozal Beylerli, Narasimha M. Beeraka, Ilgiz Gareev, Valentin Pavlov, Guang Yang, Yanchao Liang, Gjumrakch Aliev

**Affiliations:** 1 Central Research Laboratory, Bashkir State Medical University, 450008 Ufa, Republic of Bashkortostan, Russia; obeylerli@mail.ru (O.B.); ilgiz_gareev@mail.ru (I.G.); pavlov@bashgmu.ru (V.P.); 2 Department of Biochemistry, JSS Academy of Higher Education & Research, CEMR lab, DST-FIST Supported Department and Center, Mysuru 570015, Karnataka, India; bnmurthy24@gmail.com; 3 Department of Neurosurgery, the First Affiliated Harbin Medical University, Harbin 150001, China; liangyanchao@hrbmu.edu.cn; 4 Institute of Brain Science, Harbin Medical University, Harbin 150001, China; 5 Sechenov First Moscow State Medical University Sechenov University, 119146 Moscow, Russia; 6 Research Institute of Human Morphology, Russian Academy of Medical Science, 117418 Moscow, Russia; 7 Institute of Physiologically Active Compounds, Russian Academy of Sciences, Chernogolovka, 142432 Moscow, Russia; 8 GALLY International Research Institute, 7733 Louis Pasteur Drive, #330, San Antonio, TX 78229, USA

In the original publication [1], the following three references, [26,74,76], should be replaced with new references. References [26] and [74] were replaced because the articles were retracted, and reference [76] was replaced because of a citation error. With this correction, the order of some references, the contents of Table 1, and the third paragraph of Section 4 in the article have also been modified accordingly. The authors state that the scientific conclusions are unaffected. This correction was approved by the Academic Editor. The original publication has also been updated.

The corrected references that replaced references [26], [74], and [76] are as follows:

26. Zhu, D.; Xiao, Z.; Wang, Z.; Hu, B.; Duan, C.; Zhu, Z. MEG3/MIR-376B-3P/HMGA2 axis is involved in pituitary tumor invasiveness. *J. Neurosurg*. **2020**, *134*, 499–511. https://doi.org/10.3171/2019.10.JNS191959.
74. Shahrzad, J.; Monsalves, E.; Tateno, T.; Zadeh, G. Role of mTOR inhibitors in growth hormone-producing pituitary adenomas harboring different FGFR4 genotypes. *Endocrinology* **2016**, *157*, 3577–3587.
76. Bin, H.; Mao, Z.; Du, Q.; Jiang, X.; Wang, Z.; Xiao, Z.; Zhu, D.; Wang, X.; Zhu, Y.; Wang, H. miR-93-5p targets Smad7 to regulate the transforming growth factor-β1/Smad3 pathway and mediate fibrosis in drug-resistant prolactinoma. *Brain Res. Bull.* **2019**, *149*, 21–31.

The corrected version of Table 1 is as follows:

**Table 1:** Experimental information on miRNAs involved in the tumorigenesis of pituitary adenomas and their target genes by tumor type.

miRNA	Gene Target	Type of PA	Biological Function	Regulation	Phenotype	Ref.
miR-524-5p	PTTG1/PBF	NFA	Inhibition of tumor cell proliferation, migration, and invasion	Down	Tumor suppressor	[19]
miR-424, miR-503	CDC25A	NFA	Inhibition of tumor cell growth	Down	Tumor suppressor	[20]
miR-34	AIP	GH	Induces invasive properties in tumor cells	Up	OncomiR	[21]
miR-410	CCNB1	FSH/LH	Increased expression of cyclin A and D protein affecting the G1-S phase of the cell cycle; inhibition of tumor cell proliferation	Down	Tumor suppressor	[22]
miR-26a	PLAG1	NFA, GH, ACTH, PRL	Induces invasive properties in tumor cells	Up	OncomiR	[23]
miR-23b	HMGA2	NFA, FSH/LH, GH	Inhibition of tumor cell proliferation, delaying cell division in the G1 phase of the cell cycle	Down	Tumor suppressor	[24]
miR-130b	CCNA2	NFA, FSH/LH, GH	Inhibition of tumor cell proliferation, delaying cell division in the G2 phase of the cell cycle	Down	Tumor suppressor	[24]
miR-106b	PTEN	Invasive (NFA, GH, ACTH, PRL)	Induces invasive properties in tumor cells	Up	OncomiR	[25]
MIR-376B-3P with MEG3	HMGA2	Clinical nonfunctioning pituitary adenomas (CNFPAs)	HMGA2, acting as a target gene of MIR-376B-3P, functions as an oncogene in PA and can be downregulated by MEG3 through the accumulation of MIR-376B-3P	-	Tumor suppressor	[26]
miR-132, miR-15a, miR-16	SOX5	Invasive (NFA, GH, ACTH, PRL)	Inhibition of tumor cell proliferation, migration, and invasion	Down	Tumor suppressor	[27]
miR-200c	PTEN	PRL	Tumor cell apoptosis reduction	Up	OncomiR	[28]
miR-329, miR-300, miR-381, miR-655	PTTG1	PRL, GH	Inhibition of tumor cell growth	Down	Tumor suppressor	[29]
miR-20a, miR-17-5p	PTEN, TIMP2	NFA	Carcinoma metastasis	Up	OncomiR	[27]
miR-106b	PTEN-PI3K/AKT	NFA	Activation of invasive properties and migration of tumor cells; carcinoma metastasis	Up	OncomiR	[27,30]
miR-183	KIAA0101	PRL	Inhibition of tumor cell proliferation	Down	Tumor suppressor	[31]
miR-15, miR-16, miR-26a, miR-196a2, Let-7a	HMGA1, HMGA2	GH, PRL	Inhibition of tumor cell proliferation	Up	Tumor suppressor	[32]

NFA, nonfunctioning adenoma; GH, growth hormone-secreting adenoma; FSH, follicle-stimulating hormone adenoma; LH, luteinizing hormone-secreting adenoma; ACTH, adrenocorticotropic hormone-secreting adenoma; PRL, prolactin-secreting adenoma; PTTG1IP, pituitary tumor-transforming 1 interacting protein; PBF, pituitary tumor-transforming gene binding factor; CDC25A, cell division cycle 25 A; AIP, aryl hydrocarbon receptor-interacting protein; CCNB1, G2/mitotic-specific cyclin-B1; PLAG1, pleomorphic adenoma gene 1; HMGA1, high-mobility group AT-hook 1; HMGA2, high-mobility group AT-hook 2; CCNA2, cyclin A2; PTEN, phosphatase and tensin homolog deleted on chromosome 10; AKT3, serine/threonine kinase 3; SOX5, SRY-related HMG-box; SSTR2, somatostatin receptor type 2; TIMP2, tissue inhibitor of metalloproteinases 2; PI3K, phosphoinositide 3-kinases; KIAA0101, protein; miR, microRNA; PA, pituitary adenoma.

The correct contents of the third paragraph of Section 4 are as follows:

mTOR is a serine–threonine specificity protein kinase that controls cell growth and metabolism in response to various substances, growth factors, and cell stress [71]. As a central regulator of cell growth, mTOR plays a key role in the development and aging of cells and is involved in the pathogenesis of cardiovascular diseases, endocrine diseases, metabolic disorders, and tumors [72]. Antiproliferative responses to mTOR inhibition have been reported in studies with aggressive PA in vitro and in vivo [73,74]. mTOR mRNA expressions significantly modulated in invasive pituitary adenoma tissues were compared with those in noninvasive pituitary adenoma tissues [73,74].
